# Supplementary material for: Untargeted Metabolomic Assay of Prefrail Older Adults after Nutritional Intervention
Source: Metabolites. 2022 Apr 21;12(5):378. doi: 10.3390/metabo12050378 (PMC9145750; doi:10.3390/metabo12050378)
Supplement: Supplementary file 1 [file metabolites-12-00378-s001.zip › Supplementary Table S1.pdf]

Supplementary Table S1

Compounds selected and subjected to further statistical analysis after an untargeted metabolomic analysis of blood serum samples.

| | Compound | m/z | RT [min] | Analysis mode |
|---|---|---|---|---|
| 1 | Vitamin A | 287.23588 | 22.18 | PFP positive |
| 2 | Theophylline | 181.07193 | 8.49 | |
| 3 | Taurine | 126.02203 | 1.07 | |
| 4 | Stachydrine | 144.10187 | 1.45 | |
| 5 | Serotonin | 177.10216 | 8.09 | |
| 6 | Pipercitine | 350.34143 | 22.71 | |
| 7 | Myristic acid | 211.20560 | 24.08 | |
| 8 | Methylxanthine | 167.06637 | 1.46 | |
| 9 | L-Proline | 116.07087 | 1.74 | |
| 10 | Hypaphorine | 247.14395 | 11.29 | |
| 11 | **Creatinine** | 114.06643 | 2.76 | |
| 12 | **Creatine** | 132.07686 | 1.78 | |
| 13 | Alanylleucine | 203.13906 | 15.76 | |
| 14 | 3,4-Diaminopyridine | 110.07157 | 1.76 | |
| 15 | 1-Linoleoyl-sn-glycero-3-phosphocholine | 520.33964 | 18.19 | |
| 16 | 1-Hexadecanoylpyrrolidine | 310.31018 | 22.34 | |
| 17 | Lupinine | 170.15398 | 22.52 | |
| 18 | Stearic acid | 283.26428 | 23.12 | PFP negative |
| 19 | Oleic acid | 281.24860 | 22.37 | |
| 20 | L-Palmitoylcarnitine | 398.32718 | 20.09 | |
| 21 | L-Ergothioneine | 228.08080 | 1.46 | |
| 22 | Cholic acid | 407.28088 | 15.16 | |
| 23 | Tyramine | 120.08070 | 1.48 | HILIC positive |
| 24 | Phenelzine | 336.21622 | 1.19 | |
| 25 | Hypoxanthin | 137.04512 | 1.75 | |
| 26 | Dulcin | 402.21506 | 1.44 | |
| 27 | **Creatinine** | 114.06835 | 1.69 | |
| 28 | **Creatine** | 132.07675 | 6.96 | |
| 29 | 1-arachidonoyl-sn-glycero-3-phosphocholine | 520.33929 | 2.27 | |
| 30 | 1-arachidonoyl-sn-glycero-3-phosphocholine | 544.33963 | 2.19 | |
| 31 | Oleuropein | 539.17627 | 3.89 | HILIC negative |
| 32 | Brassylic acid | 243.15981 | 4.07 | |
| 33 | Amylose | 389.13882 | 8.76 | |

RT – retention time, compounds in bold were present in different analysis mode
